# Enhancement of Dissymmetry Factors Through Axial Elongation of Hetero[*n*]helicenes

**DOI:** 10.1002/anie.2703281

**Published:** 2026-08-14

**Authors:** Jeppe S. Mogensen, Christoffer Bræstrup, Christoffer Warming, Georg I. Junker, Yann Lie, Mathias Hermann, Kurt V. Mikkelsen, Michael Pittelkow

**Affiliations:** ^1^ Department of Chemistry University of Copenhagen Copenhagen Denmark

**Keywords:** benzofuran scaffolding, chirality, *g*_lum_, helicenes, structure–property relationship

## Abstract

Helicenes are attractive emitters of circularly polarized light, yet strategies to enhance their luminescence dissymmetry factor (*g*_lum_) remain underdeveloped. Herein, we report a scalable, facile synthetic route to hetero[9]helicenes utilizing a benzofuran scaffolding strategy. This efficient one‐pot protocol provides access to the hetero[9]helicenes at gram scale, enabling progressive axial elongation to hetero[12]‐ and hetero[15]helicenes. Structural characterization by NMR and single‐crystal X‐ray diffraction confirms increasing helical distortion and reduced helical pitch upon elongation. Chiroptical investigations reveal a pronounced enhancement of both absorption (*g*_abs_) and emission (*g*_lum_) dissymmetry factors with increasing ring count. Notably, annulation with a three‐ring extension via a naphtofuran unit yields a twofold increase in *g*_lum_, reaching 1.95 × 10^−2^ at 475 nm for the [15]helicene. TD‐DFT calculations reproduce these trends, revealing that the enhancement originates from a systematic decrease in the electric transition dipole moment while the magnetic transition dipole moment remains constant, thereby increasing the relative magnetic contribution to the S_1_^→^S_0_ transition as well as the angle between the dipole moments. These findings establish axial elongation as a robust design principle for amplifying chiroptical responses in helicenes.

## Graphical abstract

A benzofuran scaffolding strategy enables the synthesis of hetero[9]‐, [12]‐, and [15]helicenes. Structural and chiroptical analyses demonstrate that systematic axial elongation increases helical distortion and significantly amplifies luminescence dissymmetry. This results in a significant increase in *g*_lum_, reaching 1.95 × 10^−2^, driven by a systematic decrease in the electric transition dipole moment.

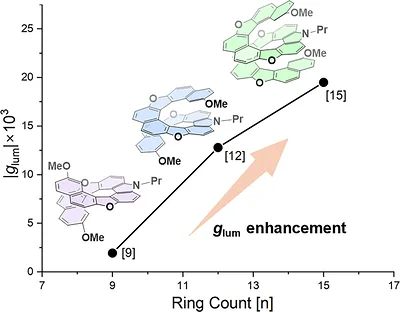

## 1 Introduction

Helicenes are a unique class of molecules consisting of *ortho*‐annulated aromatic rings forming a chiral, helical‐shaped molecular structure [1, 2, 3, 4, 5]. Their helical chirality coupled with their π‐conjugated aromatic system makes them strong candidates as intense emitters of circularly polarized light with applications within optoelectronics, CPL‐OLEDs (circularly polarized luminescence‐organic light‐emitting diodes), and bioimaging [6, 7, 8]. Despite their potential, utilization of helicenes in chiral optical applications requires enhancement of their CPL output [9, 10]. Therefore, a central challenge in the field of helicenes is enhancing the luminescence dissymmetry factor (*g*_lum_), which quantifies the emission of circularly polarized light. The highest reported *g*_lum_ values for helicenes are in the order of ∼10^−2^ [11, 12]. Studies correlating *g*_lum_ with systematic structural modification remain scarce. Consequently, no general structural design principles for *g*_lum_ optimization have been formulated, further complicating the enhancement of *g*_lum_ [13, 14]. Axial elongation has emerged as a promising pathway toward the enhancement of *g*_lum_ [15, 16]. For an electronically allowed transition, the *g*_lum_ depends on three parameters: the electronic ($\vec{\mu }$), magnetic ($\vec{m}$) transition dipole moments for the S_1_ → S_0_ transition, and the angle between them θ. These parameters are related by the formula: *g*_lum_ ≈ $4\;(|\vec{m}\left|\right|\vec{\mu }|)\cdot \cos\theta$ [6]. Theoretical studies have indicated that helicenes behave similarly to a nano‐solenoid in which increasing the number of turns in the coil leads to an increase in the magnetic moment and therefore an increase in *g*_lum_ [11, 17]. It is still uncertain whether this correlation between turn numbers and increased *g*_lum_ holds. Several studies support the correlation (Figure 1), such as the tetraaza[*n*]helicenes (*n* = 7, 9, 11, 13, 15) reported by Shibata [18], the expanded‐aza[*n*]helicenes (*n* = 11, 19, 27, 35, 43) reported by Wu [19], PDI‐fused helicenes NP[*n*]H (*n* = 2, 3, 4, 5) reported by Nuckolls [20, 21] or the extended [*n*]helicenes (*n* = 7, 9) reported by Narita [22]. However, several other studies [23, 24, 25] such as the fully *ortho*‐annulated aza[*n*]helicenes (*n* = 9, 11, 13, 15, 17, 19) reported by Tanaka [26] show no direct correlation between helical length and *g*_lum_, highlighting the need for studies which correlate ring count with predictable changes in chiral optical properties. Further, understanding the effect of axial elongation requires effective scalable protocols toward axially elongated helicenes [27]. Axial elongation of helicenes is associated with an increase in steric strain, rendering it synthetically challenging [28, 29, 30, 31], making the development of effective scalable protocols for axial elongation of particular interest.

**FIGURE 1:**
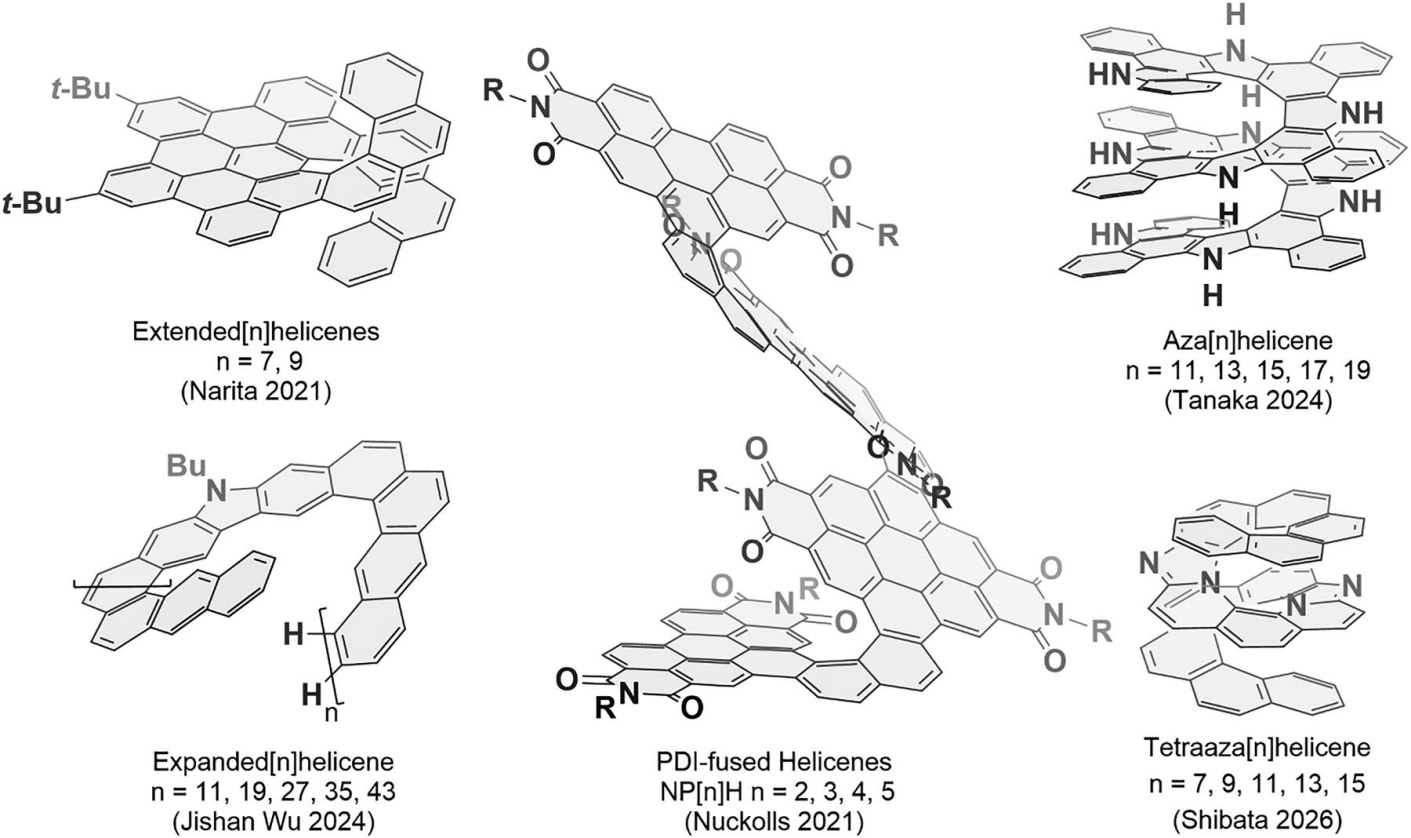
Selected examples of homologous series of helicenes reported in literature: extended[*n*]helicenes, PDI‐fused helicenes, expanded[*n*]helicenes, aza[*n*]helicenes, and tetraaza[n]helicenes.

We have previously utilized the inherent chemistry of phenols in a benzofuran scaffolding approach [32] to obtain a series of hetero[*n*]helicenes (*n* = 7, 10, 13) [33].

This approach relies on an initial oxidative C–C coupling of the *ortho*‐position relative to the phenol followed by an intramolecular acid‐mediated dehydration. We have also established a facile synthetic route to hetero[9]helicenes, albeit with significant oxidative byproduct formation [34]. In this study, we describe a scalable and optimized synthetic route to hetero[9]helicenes. We further report the synthesis of hetero[*n*]helicenes (*n* = 12, 15) via a systematic elongation of the [9]helicenes using a benzofuran scaffolding approach. We showcase significant enhancement of *g*_lum_ and *g*_abs_ upon elongation, providing insight into possible design principles for chiroptical enhancement of helicenes. We also report quantum chemical simulations of the absorption and emission spectra, along with calculations of the electronic and magnetic transition dipole moments for the presented hetero[*n*]helicenes.

## 2 Results and Discussions

We began our investigations by reexamining our previously described synthesis of [9]helicenes [34]. The benzofuran scaffolding strategy is a 2‐step‐1‐pot protocol, where an oxidative C–C coupling is followed by an acid‐mediated dehydration to form a furan. The oxidative coupling between carbazole **1** and naphthol **2** using chloranil in chlorobenzene quantitatively yielded compound **A** within 8 h (Scheme 1). In previous work, the combination of BF_3_•OEt_2_ and chloranil promoted the desired dehydration but also produced oxidized byproducts. Therefore, a non‐oxidizing acid was chosen in the furan‐forming dehydration step. Without work‐up, direct addition of 4 equiv of *p*‐toluene sulfonic acid monohydrate (TsOH•H_2_O) into the reaction mixture and boiling under reflux resulted in the formation of helicene **3a** (67% yield). This protocol also led to the loss of *tert*‐butyl groups on the carbazole moieties. The loss of the *tert*‐butyl groups did not result in significant reduction of solubility, likely due to the non‐planar structure of the molecules. Increasing the amount of TsOH•H_2_O to 6 equiv promoted *O*‐demethylation, in addition to ring closure and removal of *tert*‐butyl groups, yielding helicene **3b** (24%) and helicene **3c** (5%) along with **3a** (40%). Demethylation of helicene **3a** was desired as the hydroxy‐[9]helicenes **3b** and **3c** were targeted for further axial elongation. These unanticipated demethylation and de‐*tert*‐butylation reactions resulted in a synthetic procedure with double oxidative coupling, dehydration, reverse‐Friedel–Crafts alkylation, and demethylation taking place within a single reaction vessel. The reaction proved reliable at the gram scale, providing a very efficient synthetic route to hetero[9]helicenes.

**SCHEME 1:**
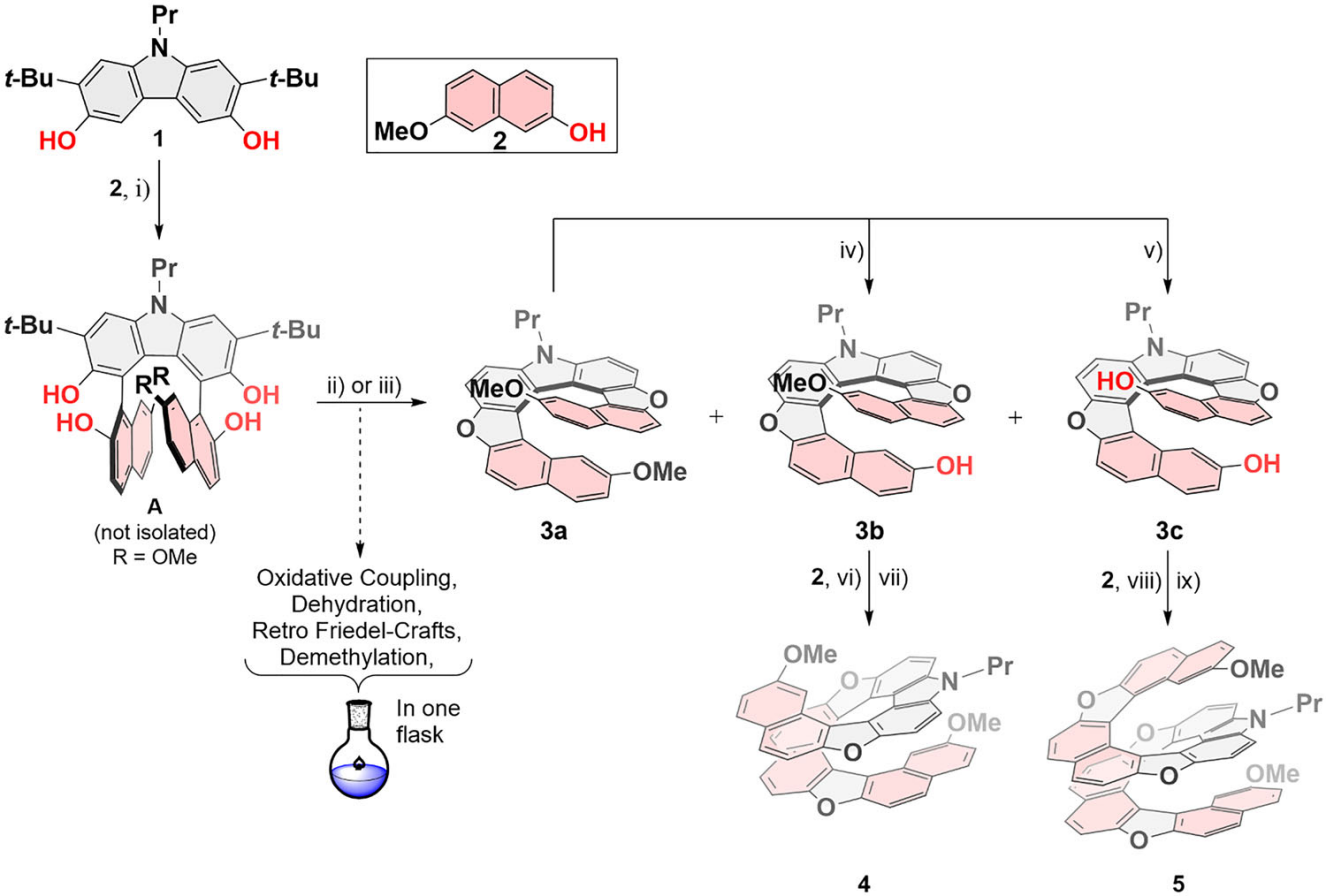
Synthetic route to helicenes **3a**, **3b**, **3c**, **4**, and **5**. (i) Chloranil (2 equiv.), PhCl, r.t., 8 h, (ii) TsOH•H_2_O (4.4 equiv.), PhCl, reflux, 69 h, 67%. (iii) TsOH•H_2_O (6 equiv.), PhCl, reflux, 64 h, 40% (**3a**), 24% (**3b**), 5% (**3c**). (iv) BCl_3_ (1.8 equiv.), TBAI (1 equiv.), CH_2_Cl_2_, r.t., 3 h, 33%. (v) BCl_3_ (10 equiv.), TBAI (4 equiv.), CH_2_Cl_2_, r.t., 3 h, 70%. (vi) Cu^II^Cl(OH)‐TMEDA (2.5 equiv.), PhCl, air, r.t., 16 h. (vii) TsOH•H_2_O (10 equiv.), PhCl, 100°C, 6 h, 18% (4). (viii) Cu^II^Cl(OH)‐TMEDA (2.2 equiv.), PhCl, air, r.t., 46 h. (ix) TsOH•H_2_O (10 equiv.), PhCl, 100°C, 3 h, 6% (5).

Lewis acid mediated demethylation of **3a** provided another straightforward pathway to helicenes **3b** and **3c**. While previously reported [9]helicenes carrying both *tert*‐butyl groups on the [9]helicene underwent demethylation with BBr_3_ within 3 h, demethylation of helicene **3a** was significantly slower and required the combination of BCl_3_ and TBAI for full demethylation within 3 h [35]. Further, axial elongation from the [9]helicenes required the use of an oxidant which could selectively oxidize the terminal naphthol at the 1‐position adjacent to the phenol. Chloranil in PhCl did not show any reactivity, and the focus was therefore turned toward first row transition metal catalysts, which have typically been used in the analogous formation of BINOLs [36]. Treating [9]helicene **3b** and naphthol **2** with Cu^II^Cl(OH)‐TMEDA (TMEDA = *N*,*N*,*N^’^*,*N^’^*‐tetramethylethylenediamine) resulted in complete consumption of both starting materials. Subsequent addition of TsOH•H_2_O and refluxing resulted in the formation of [12]helicene **4**. Subjecting helicene **3c** to analogous synthetic conditions resulted in the formation of [15]helicene **5**, consisting of 15 *ortho*‐annulated fully conjugated aromatic rings. Although the synthesis of helicenes **4** and **5** proceeded in low yield, the reaction procedure was robust and reproducible. Curiously, *O‐*demethylation, as observed for **3** under treatment with TsOH•H_2_O, was not observed for either **4** or **5** (Scheme 1).

All synthesized helicenes (**3a**, **3b**, **3c**, **4**, **5**) were fully characterized by ^1^H‐NMR, ^13^C‐NMR spectroscopy, HRMS, and X‐ray crystallography. Notably, the CH_2_ propyl protons (closest to the nitrogen) of helicenes **4** and **5** display diastereotropic behavior and fully separate into two distinct signals. The methoxy groups of helicenes **4** and **5** at 2.71, 2.81, and 2.33 ppm, respectively, in CD_2_Cl_2_ are significantly upshifted compared to the methoxy of naphthol **2** at 3.90 ppm in CDCl_3_ [37], due to the shielding effect of the aromatic ring positioned directly above and below the methoxy groups. The X‐ray crystal structures confirmed the helical structure of all the helicenes. Hydrogen bonding from the ─OH to solvent molecules was observed for helicenes **3b** and **3c**. Crystals suitable for X‐ray diffraction were obtained through slow evaporation of mixtures of *i*‐PrOH and CH_2_Cl_2_ for both helicenes **4** and **5**. The helical pitch of **3a**, **4**, and **5** measured from centroid to centroid of overlapping benzene rings (Figure 2) decreases with increased helicene length, revealing that the helical framework becomes more compact as it extends. The increase in helical distortion is also observed from the torsion angles of the central pyrrole ring. The torsion angles of the central pyrrole ring increase along the series **3a** (16.8°), **4** (20.8°), **5** (24.5°) (Table S2), while the inner furan of helicenes **4** (4.18°) and **5** (5.06°) remain almost planar. This is similar to our previously reported hetero[n]helicene (n = 7, 10, 13) series in which the central heterocyclic ring also accommodates most of the bend.

**FIGURE 2:**
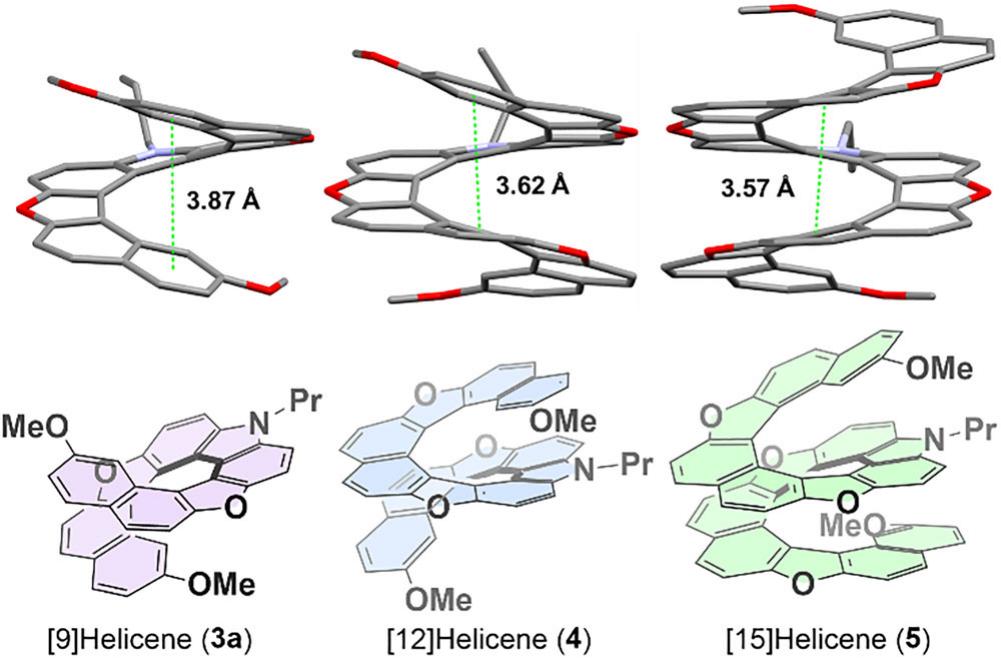
Top: X‐ray crystal structures and helical pitch of helicenes **3a**, **4**, and **5**. C: Grey, O: red, N: blue. Hydrogens and solvent molecules omitted for clarity. Bottom: Structures of helicenes and color coding for the discussion of the chiral optical properties.

The absorption and fluorescence spectra of helicenes **3a**, **4**, and **5** revealed a small redshift (18 nm) upon increasing the size of the helical system. Despite increasing the π‐system, a decrease in molar extinction coefficient (*ε*) was also observed upon increased helical length. This decrease is likely caused by the non‐planar helical structure, which leads to a decrease in the effective conjugation of the helicenes. The Stokes shift increases from 1338 cm^−1^ (**3a**) to 1778 cm^−1^ (**5**) (Table 1) with increased length of the helicenes, possibly due to more non‐radiative decay pathways with increased flexibility in the helicenes. The quantum yields (*Φ*) for helicenes **3a**, **4**, and **5** were determined to be 0.51, 0.18, and 0.10, respectively, showing a decrease with more annulated rings as observed for other series of helicenes [18, 26, 33]. The decrease in quantum yield is also reflected in the increase in the non‐radiative rate constant, which more than doubles from **3a** (0.118 ns^−1^) to **5** (0.259 ns^−1^), possibly also due to increased flexibility in the longer helicenes. The enantiomers of helicenes **3a**, **4**, and **5** were separated using semi‐preparative recycling HPLC on a chiral stationary phase. The ECD and CPL of helicenes **3a**, **4**, and **5** measured in CH_2_Cl_2_ (Figure 3) revealed symmetrical absorption and emission peaks for the enantiomers, confirming the enantiomeric purity. The ECD revealed a negative Cotton effect for helicene **3a** in the highest wavelength absorption. TD‐DFT enabled us to assign the absolute configuration of the helicenes **3a**, **4**, and **5** (Figures S50 and S51). The *P*‐enantiomers for helicenes **3a**, **4**, and **5** give rise to a positive lobe in the most redshifted absorption. Here, the effect of elongation is pronounced as the *g*_abs_ increases substantially with length for almost all wavelengths. A similar trend is observed for the *g*_lum_ values (Table 1). Remarkably, annulation with a single naphthol unit leads to almost twofold enhancement of *g*_lum_. The [15]helicene (**5**) exhibits *g*_lum_ of 1.95 × 10^−2^ at 475 nm. It should be noted that even though axial elongation for this series of helicenes increases *g*_lum_ across the series, the overall CPL‐brightness (Table S3) is optimal for the [12]helicene (**4**), highlighting the delicate balance required when optimizing chiroptical properties.

**TABLE 1:** Summary of the chiral optical properties of helicenes **3a**, **4**, and **5**.

Helicene	λ_max _(nm)	*ε* (м^−1^ cm^−1^)^a^	Stokes shift (cm^−1^)	*Φ* (%)	|*g*_lum_|	|*g*_abs_|	Non‐radiative rate *k*_nr_ (ns^−1^)
[9] (**3a**)	420	24 000 (±711)	1338	51	1.94 × 10^−3^ at 430 nm	1.84 × 10^−3^	0.118
[12] (**4**)	429	13 900 (±711)	1380	18	1.28 × 10^−2^ (459 nm)	4.89 × 10^−3^	0.195
[15] (**5**)	438	6200 (±105)	1778	10	1.95 × 10^−2^ (475 nm)	8.51 × 10^−3^	0.259

*Note*: Numbers in brackets represent standard deviation from triplicate measurements.

**FIGURE 3:**
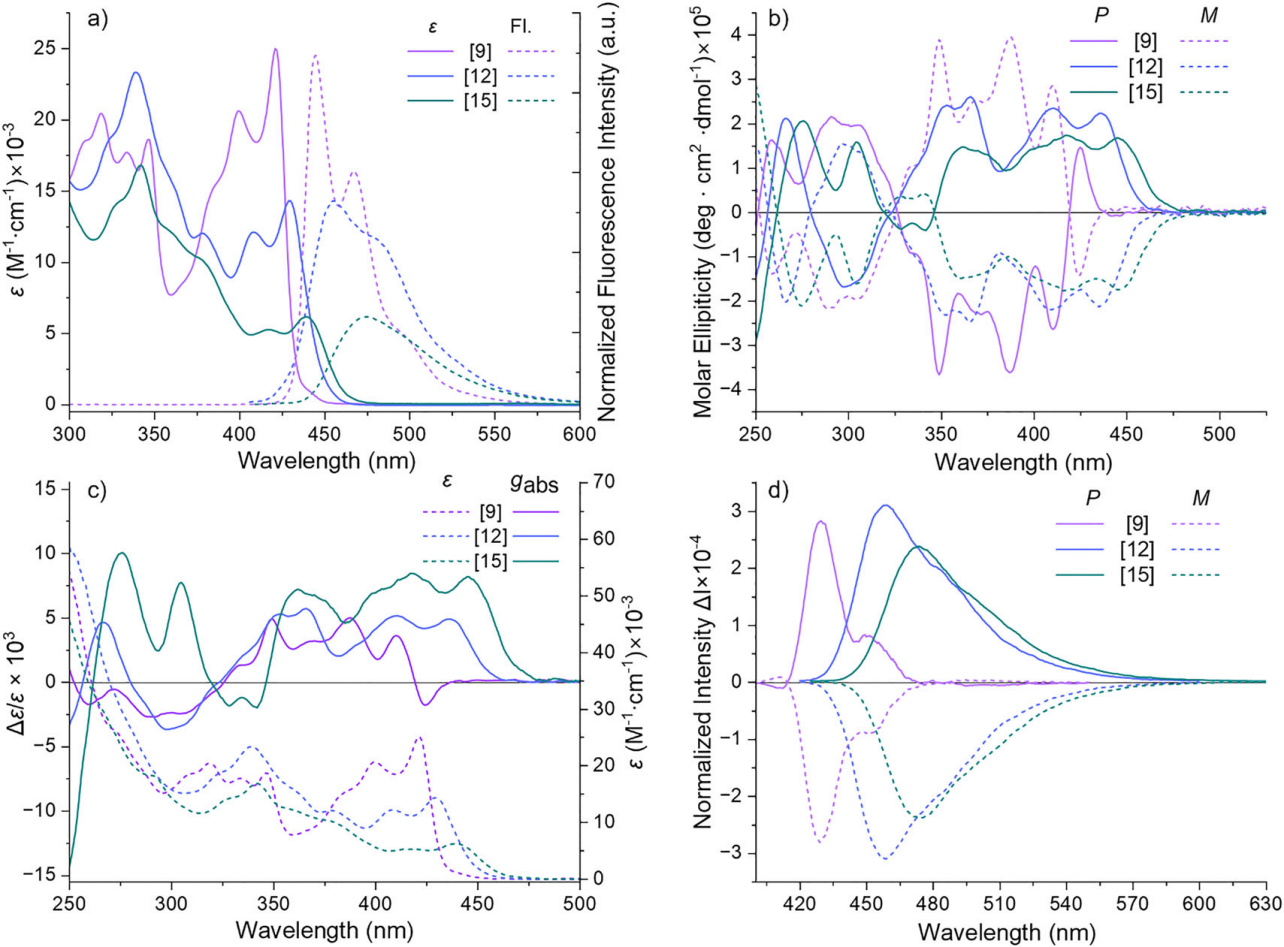
(a) Absorbance and fluorescence spectra for helicenes **3a** [9], **4** [12], and **5** [15] in CH_2_Cl_2_. Excitation at: 420 nm (**3a**), 380 nm (**4**), 380 nm (**5**), (b) ECD spectra of helicenes **3a**, **4**, and **5** in CH_2_Cl_2_. (c) Absorbance and *g*_abs_ spectra of helicenes **3a**, **4**, and **5**. (d) CPL spectra of helicenes **3a**, **4**, and **5**. Excitation at 345 nm (**3a**), 338 nm (**4**), 333 nm (**5**).

Having established a clear increase in *g*_lum_ upon annulation with naphthalene units, we wondered whether helical length is the primary factor governing *g*_lum_ for these molecules. To examine this, we compared the *g*_lum_ as a function of helical length [*n*] for our newly synthesized series of helicenes and previously synthesized helicenes from our group, which also consist of naphthol and carbazole motifs with diverse functionalization at both the terminal oxygens and the aromatic core (Figure 4). For these molecules, elongation of the helical framework consistently results in enhanced *g*_lum_ values. Notably, despite the electronic differences, the functionalization of the helicene core exerts only a marginal impact on the dissymmetry factor. Compound **4** differs from compounds **3a** and **5**. It has the carbazole unit positioned closer to one end of the helicene rather than near the center of the helical framework. Despite this structural difference, compound **4** follows the same monotonic increase in both *g*_abs_ and *g*_lum_. This showcases axial elongation of the helicene core as the main structural determinant of *g*_lum_ for this series of molecules. We further compared the *g*_lum_ trajectory with helical length for our newly synthesized helicenes and the homologous series of helicenes shown in Figure 1. Excluding the aza[*n*]helicenes, an increase in *g*_lum_ is observed with increased helical length. The longest member of each series displays the highest *g*_lum_ value, resulting in *g*_lum_ around 10^−2^ (Figure S49). Our series of helicenes displays an outstanding trajectory in *g*_lum_, which has only been observed in the tetraaza[*n*]helicenes reported by Shibata and coworkers [18]. To understand the origin of *g*_lum_ enhancement in this series of helicenes, we undertook investigations into the transition dipole moments using TD‐DFT calculations with the functional CAM‐B3LYP‐D4 [38, 39] and the def2‐SVP [40] basis set as implemented in ORCA [41, 42]. Solvation of the helicenes was modeled using C‐PCM [43] (see Section 6 in Supporting Information for further computational details).

**FIGURE 4:**
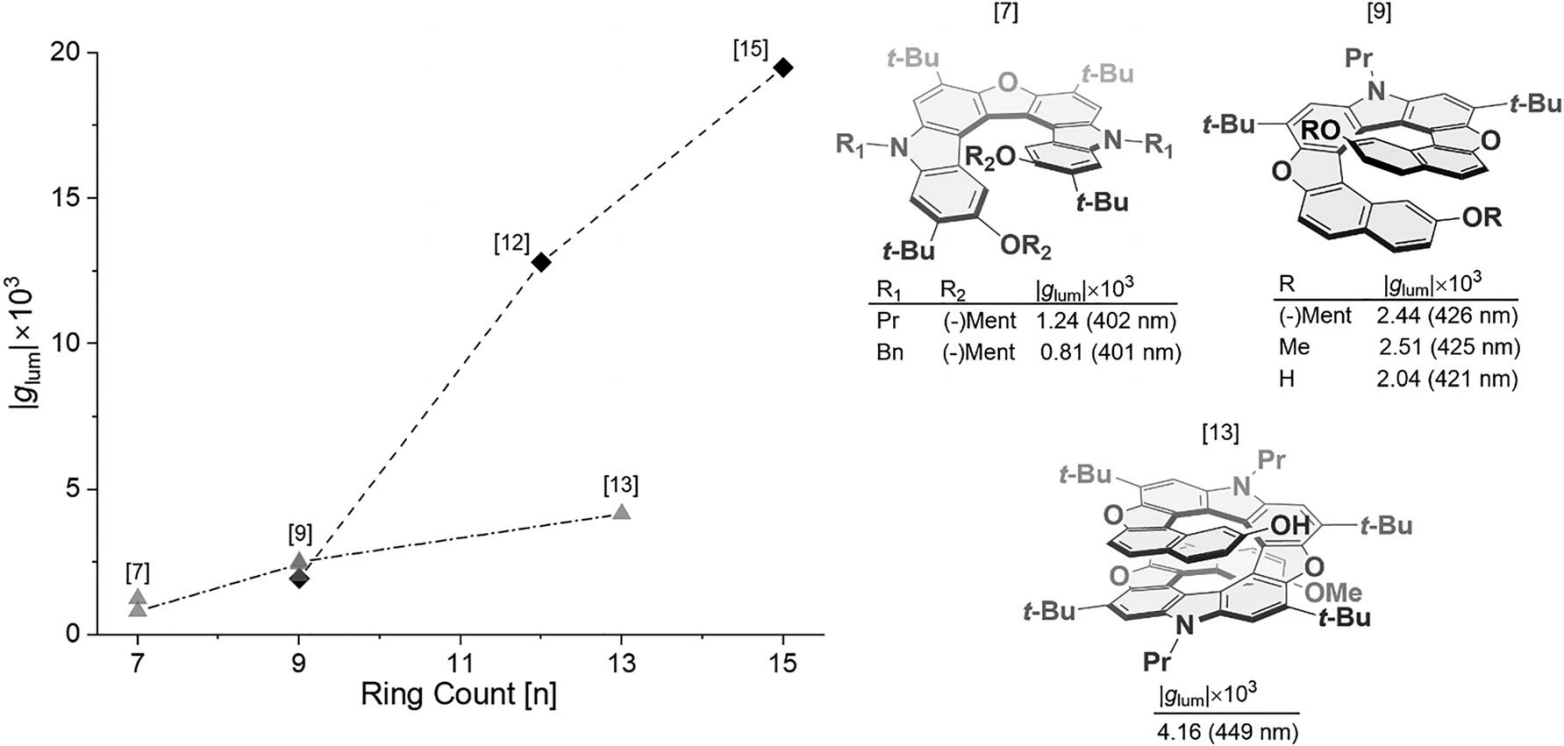
Plot of |*g*_lum_| as a function of ring count [*n*] of the helicenes synthesized in the Pittelkow group. All spectra were measured in CH_2_Cl_2_. 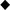 = helicenes **3a**, **4**, and **5**, 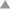 = [7], [9], [10], and [13] helicenes and structures of [7], [9] [34], and [13] [44] helicenes synthesized in the Pittelkow group and used in the plot. (−)Ment: ─CO‐(−)menthol.

We note that the calculated CPL and ECD transitions match the experimentally recorded spectra, with a slight blueshift of the absorbance and emission maxima (Figures S50 and S51). Two effects are responsible for the increase in *g*_lum_ values upon elongation (Figure 5). First, the magnitude of the electronic transition dipole moment decreases along the series, while the magnitude of the magnetic transition dipole moment remains constant, leading to an increase in the $|\vec{m}\left|/\right|\vec{\mu }|$ ratio. Secondly, the angle between the transition dipole vectors increases upon elongation. This results in the transition dipole moments becoming more antiparallel, contributing to a larger dissymmetry factor. Although compound **4** possesses a different placement of the carbazole unit compared with compounds **3a** and **5**, the calculated transition dipole moments exhibit the same systematic evolution observed across the homologous series. This indicates that the global electronic structure of the extended helicene framework dominates the optical transition, while the different carbazole position introduces only a local perturbation that does not alter the overall mechanism responsible for the enhanced *g*_lum_.

**FIGURE 5:**
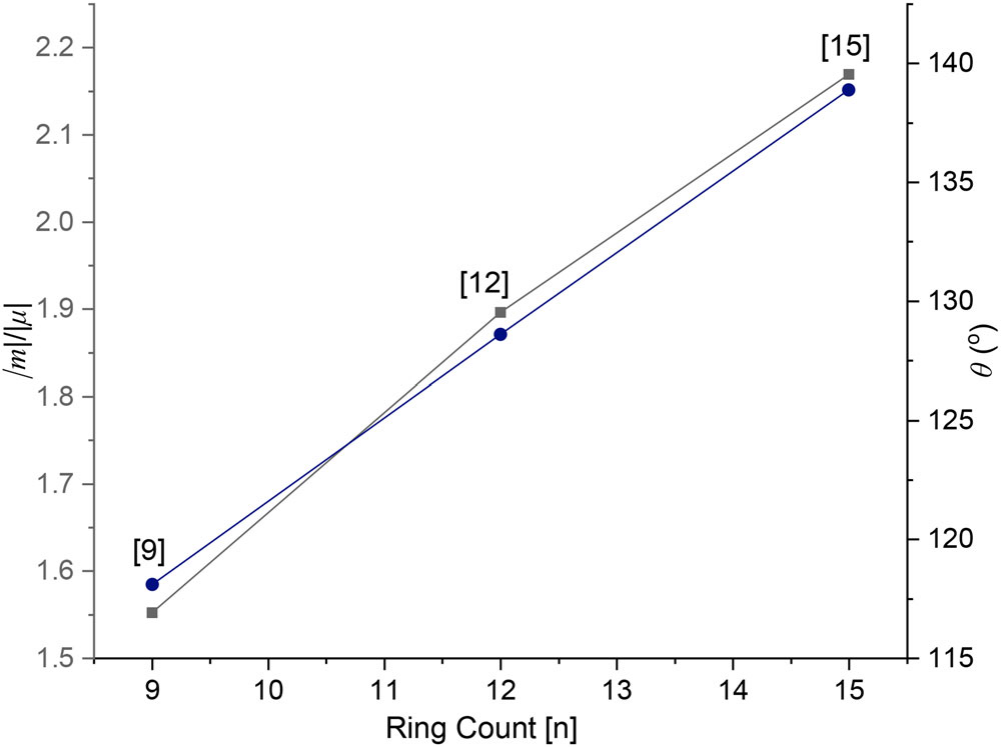
Calculated $|\vec{m}\left|/\right|\vec{\mu }|$ (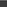) and θ (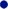) for helicenes **3a**, **4**, and **5** as a function of ring count.

The direction of the magnetic transition dipole vector for all helicenes is aligned along the helical axis (Figure 6, red vectors). This alignment indicates that the helicenes are behaving as a nano‐solenoid. However, the magnetic transition dipole moment does not increase across the series, suggesting the electron behavior of the helicenes to be more complex.

**FIGURE 6:**
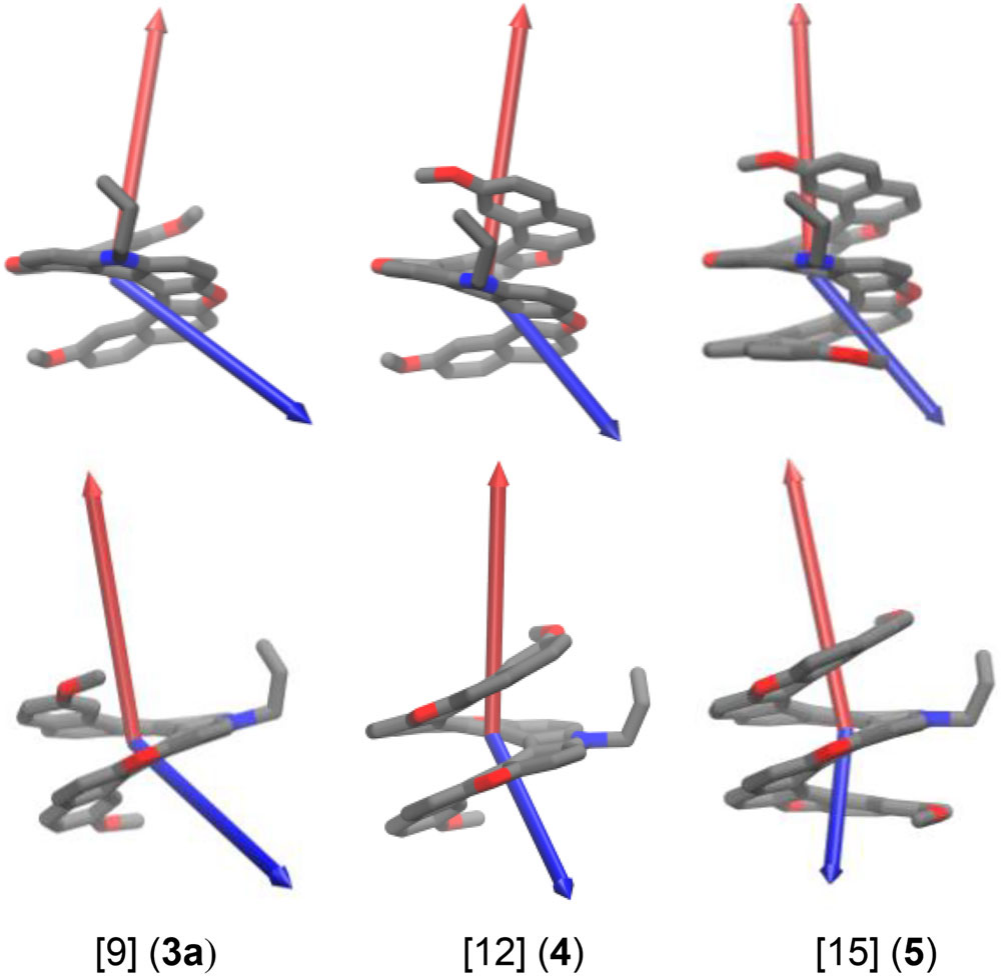
Illustration of the orientation of the calculated transition dipole vectors for the fluorescence of helicenes **3a**, **4**, and **5** in CH_2_Cl_2_. Sideview: blue, μ; red, m. The TD‐DFT calculations were performed at the CAM‐B3LYP‐D4/def2‐SVP level‐of‐theory and used C‐PCM to model the solution. Note that both vectors have been scaled with a factor of 6.

## 3 Conclusion

We have developed a very efficient and scalable synthetic protocol for the synthesis of hetero[9]helicenes via benzofuran scaffolding. We further annulated the [9]helicenes systematically with one and two naphthol units, yielding hetero[*n*]helicenes (*n* = 12, 15). We obtained single x‐ray crystal structures of all helicenes and fully characterized them. We demonstrated the effect of axial elongation on the absorbance, fluorescence, and chiral optical properties, revealing a substantial increase in *g*_abs_ and *g*_lum_ as a consequence of axial elongation for this series of helicenes. TD‐DFT calculations revealed that a decrease in the magnitude of the electronic transition dipole moment is the primary contributor to the increase in chiral optical response. We have exemplified a promising path to increasing *g*_abs_ and *g*_lum_ for helicenes. This work further builds toward the formulation of design principles for optimization of chiral optical properties of helicenes, holding promise for the utility of multilayered helicenes in optical devices.

## Author Contributions

**Jeppe S. Mogensen**: methodology, investigation, formal analysis, data curation, validation, writing – original draft, writing – review and editing. **Christoffer Bræstrup**: investigation, methodology, validation, formal analysis, writing – original draft, writing – review and editing. **Christoffer Warming**: investigation, methodology, writing – review and editing. **Georg I. Junker**: data curation, formal analysis, investigation, writing – review and editing. **Yann Lie**: investigation, methodology, writing – review and editing. **Mathias Hermann**: investigation, methodology, writing – review and editing. **Kurt V. Mikkelsen**: supervision, writing – review and editing. **Michael Pittelkow**: conceptualization, investigation, funding acquisition, writing – original draft, methodology, validation, visualization, writing – review and editing, project administration, supervision.

## Conflicts of Interest

The authors declare no conflicts of interest.

## Supporting information



All synthetic and experimental details as well as computational details can be found in the Supporting Information. The authors have cited additional references within the Supporting Information [45–62].**Supporting File**: anie74224‐sup‐0001‐SuppMat.docx.

## Data Availability

The data that supports the findings of this study are available in the Supporting Information of this article.
