# Supplementary material for: Enhancement of Dissymmetry Factors Through Axial Elongation of Hetero[n]helicenes
Source: Angew Chem Int Ed Engl. 2026 Aug 14;65(41):e2703281. doi: 10.1002/anie.2703281 (PMC13638125; doi:10.1002/anie.2703281)
Supplement: Supplementary file 1 [file ANIE-65-e2703281-s001.docx]

Enhancement of Dissymmetry Factors through Axial Elongation of Hetero[n]helicenes

Jeppe S. Mogensen, Christoffer Bræstrup, Christoffer Warming, Georg I. Junker, Yann Lie, Mathias Hermann, Kurt V. Mikkelsen, Michael Pittelkow.

Department of Chemistry, University of Copenhagen, Universitetsparken 5, 2100 Copenhagen Ø, Denmark

[1. General Experimental	3](#_Toc236300204)

[2. Synthesis	5](#_Toc236300205)

[3. NMR and MS Spectra (Figure SI 1 - 28)	12](#_Toc236300206)

[4. X-Ray Crystallography	34](#_Toc236300207)

[4.1. Helicene 3a	36](#_Toc236300208)

[4.2. Helicene 3b	37](#_Toc236300209)

[4.3. Helicene 3c	38](#_Toc236300210)

[4.4. Helicene 4	40](#_Toc236300211)

[4.5. Helicene 5	41](#_Toc236300212)

[5. Optical Measurements	43](#_Toc236300213)

[5.1. Fluorescence lifetimes	43](#_Toc236300214)

[5.1.1. Helicene 5	45](#_Toc236300215)

[5.2. Chiral Chromatography	46](#_Toc236300216)

[5.3. Quantum yield measurements.	48](#_Toc236300217)

[5.4. Summary of Chiral Optical Properties	60](#_Toc236300218)

[5.5. Literature *g*_lum_ Comparison	61](#_Toc236300219)

[6. Computational Details and Spectra	62](#_Toc236300220)

[7. References	65](#_Toc236300221)

# 1. General Experimental

All chemicals were purchased from commercial suppliers and used as received. Drying of solvent was achieved with activated molecular sieves (4 Å). HPLC-grade solvents were used in all reactions. TLC was conducted on Merck-DC-Alufolien SiO_2_ 60 F254 0.2 mm thick precoated TLC plates. Melting points were measured on a Stuart SMP3 apparatus and are uncorrected. ^1^H NMR spectra were recorded on either a Bruker 500 MHz spectrometer with a non-inverse cryoprobe or an inverse cryoprobe. All ^13^C NMR spectra were recorded on a Bruker 500 MHz spectrometer with a non-inverse cryoprobe. All ^1^H-NMR and ^13^C NMR spectra are referenced to the residual solvent peak (CDCl_3_ δ_H_ = 7.26 ppm and δ_C_ = 77.16 ppm; DMSO-*d*_6_ δ_H_ = 2.50 ppm and δ_C_ = 39.52 ppm; CD_2_Cl_2_ δ_H_ = 5.32 ppm and δ_C_ = 53.84 ppm). The NMR data were processed using MestReNova v. 14.3.1-31739.

Purification by column chromatography was carried out using SiO_2_ (particle size for flash column chromatography: 40-63 μm, particle size for dry column vacuum chromatography: 15-40 μm).

UV-Vis spectra were measured on a Cary5000 double-beam spectrometer from Agilent Technologies. All UV-Vis measurements were performed in CH_2_Cl_2_ at ~293 K. The molar extinction coefficient ε was determined by weighing out 1-5 mg of compound and performing a 5-point dilution series. The molar extinction coefficients were determined as an average of 3 experiments.

Fluorescence spectra were measured on a Perkin Elmer Luminescence Spectrometer LS 50 B with a double monochromator for both detection and excitation. Fluorescence measurements were conducted at ~293 K in CH_2_Cl_2_. Fluorescence spectra were recorded from 300 nm to 800 nm in CH_2_Cl_2_ as solvent, with excitation at 380 nm for the [12] and [15]helicenes and excitation at 420 nm for the [9]helicene.

MALDI-FT-ICR HRMS was measured on a SolariX ESI/MALDI-FTMS spectrometer with dithranol as the matrix. External calibration of the spectrometer was conducted with sodium trifluoroacetate cluster ions.

CPL was measured on a JASCO CPL-300 spectrometer equipped with a JASCO PM-539 photomultiplier detector. Samples were dissolved in CH_2_Cl_2_ in a 10 mm path length quartz Suprasil (Hellma QS) cuvette and diluted until an absorbance of 0.1±0.01 at the excitation wavelength was reached. Helicene **3a** was excited at 345 nm. Helicene **4** was excited at 338 nm. Helicene **5** was excited at 333 nm. Instrument parameters for all measurements were: Scanning speed: 20 nm/min (continuous), data pitch: 0.5 nm, digital integration time: 16 s, excitation monochromator slit width: 20 nm, emission monochromator slit width: 10 nm. Photomultiplier voltage: 600 V. Spectra were accumulated from 7 (helicene **3a**) and 9 (helicene **4** and **5**) scans and *g*_lum_ calculated with the instrument software.

CD spectra were measured on a JASCO 1700 Spectrometer. All CD and CPL measurements were performed in the same cuvette. A baseline of pure solvent was recorded and subtracted from the measurements.

Chiral resolution was achieved on a JAI LaboACE LC-7080 plus recycling GPC connected with a Nacalai Tesque COSMOSIL cholester column 10 × 250 mm and a COSMOSIL Cholester guard column 10ID × 20 mm, flow rate: 2 mL/min in MeOH/CH_2_Cl_2_ (1:4) mixtures. Between 1-10 mg were injected sample and separated through multiple cycles. The number of cycles required depended on the specific compound. When a clear separation of the enantiomers was observed, the pure enantiomers were eluted from the loop. The samples were reinjected if insufficient enantiomeric purity was observed on CPL.

Quantum yields were determined using the relative method with 5-point dilution curves. A solution of the compound of interest with absorbance of around 0.1 was prepared. This solution was diluted 5 times with an arbitrary amount of solvent. For each dilution, an absorption and a fluorescence spectrum were measured. The integrated emission was plotted as a function of the absorbance at the excitation wavelength, and the slope was determined. The same dilution procedure was also conducted for the reference compound coumarin-153. All compounds and the reference were excited at 395 nm.^[45]^ Fluorescence lifetimes were measured on a FluoTime300 spectrometer from Picoquant using a laser at 390 nm.

The quantum yields were determined with the following formula:

$$\Phi_{x}=\Phi_{ref}*\frac{{Slope}_{x}}{{Slope}_{ref}}*\left( \frac{n_{x}^{2}}{n_{ref}^{2}} \right)$$

Where subscript *ref* denotes the reference compound (coumarin-153) and subscript *x* denotes the compound of interest. $\Phi$ = Quantum yield. $Slope$= slope obtained from plotting integrated emission as a function of absorbance. $n$ = refractive index of the solvent. The samples were excited at 395 nm using a PTI QuantaMaster8075 from Horiba Scientific equipped with a Xenon lamp. Coumarin-153 (Sigma-Aldrich) in pure ethanol (HPLC grade, Sigma Aldrich) $(n=1.3611)$ was used as a reference ($\Phi_{ref}=0.53)$. CH_2_Cl_2_ $(n=1.4125)$ was used as a solvent in measurements of the helicenes.

# 2. Synthesis

Compounds **1** and **2** were synthesized according to literature procedures.^[46 - 47]^

Synthesis of helicenes **3a**, **3b** and **3c**

**Procedure:** In a 100 mL flame dried round bottom flask 3,6-dihydroxy-2,7-di-*tert*-butyl-N-propyl carbazole **1**, (800 mg, 2.26 mmol, 1.00 eq.) and 2-hydroxy-7-methoxy naphthalene **2**, (788 mg, 4.53 mmol, 2.00 eq.) were dissolved in anhydrous chlorobenzene (35 mL). Chloranil (1.11 g, 4.53 mmol, 2.00 eq.) was added in one portion and the reaction mixture was stirred at room temperature for 8 hours under a nitrogen atmosphere. Then *p*-toluene sulfonic acid monohydrate (2.58 g, 13.58 mmol, 6.00 eq.) was added in one portion, and the reaction mixture was stirred under nitrogen at reflux for 64 hours. The reaction was quenched with saturated NaHCO_3_ (40 mL), thereafter extracted with CH_2_Cl_2_ (30 mL) and EtOAc (2×30 mL) with addition of absolute EtOH (15 mL) during extraction. The combined organic phases were concentrated *in vacuo* and purified by dry column vacuum chromatography (Ø: 4 cm, length: 7 cm, 40 mL fractions, toluene in *n*- heptane (v/v), 7.5% gradient increment, gradient held for 4 fractions at 60%, having reacted a gradient of 100% toluene increment changed to CH_2_Cl_2_ in toluene 7.5% (v/v), helicene **3a** elutes in fractions 13 - 16, helicene **3b** elutes in fractions 21-24, helicene **3c** elutes in fractions 27-29) The combined fractions were evaporated to dryness separately to yield helicene **3a** and **3c** as yellow solids. The remaining solid from evaporation of the fractions containing helicene **3b** was treated with saturated K_2_CO_3_ solution (30 mL) and extracted with CH_2_Cl_2_ (2×40 mL). The combined organic phases were concentrated *in vacuo* to yield helicene **3b** as a yellow solid.

Helicene **3a**:

**Yield:** 500 mg, 910 μmol, 40%.

**^1^H-NMR (500 MHz, DMSO-*d*_6_):** δ = 8.02 (d, *J* = 8.8 Hz, 2H), 7.98 (d, *J* = 8.8 Hz, 2H), 7.61-7.58 (m, 4H), 7.46 (d, *J* = 2.6 Hz, 2H), 7.40 (d, *J* = 8.8 Hz, 2H), 6.50 (dd, *J* = 8.8, 2.6 Hz, 2H), 4.75-4.66 (m, 2H), 2.95 (s, 6H), 2.05-1.95 (m, 2H), 1.06 (t, *J* = 7.4 Hz, 3H).

**^13^C-NMR (126 MHz, DMSO-*d*_6_):** δ = 155.2, 153.4, 150.3, 137.8, 129.9, 128.7, 127.7, 124.3, 120.0, 118.9, 114.3 ,114.2, 110.1, 109.1, 108.4, 106.8, 53.44, 44.65, 22.34, 11.56.

***R*_f_:** 0.14 (*n*-heptane/CH_2_Cl_2_ 1:1).

**Melting Point:** Decomposition 280 ^o^C.

**HRMS (MALDI-FT-ICR):** Calculated for C_37_H_27_NO_4_ [M**^•^**^+^] 549.1935, found 549.1929.

Helicene **3b**:

**Yield:** 300 mg, 545 μmol, 24%.

**^1^H-NMR (500 MHz, DMSO-*d*_6_):** δ = 8.60 (s, 1H), 8.01-7.94 (m, 4H), 7.59-7.55 (m, 2H), 7.52-7.50 (m, 2H), 7.47 (d, *J* = 2.2 Hz, 1H) ,7.40-7.37 (m, 2H), 7.29 (d, *J* = 8.7, 1H), 6.46 (dd, *J* = 8.9 Hz, 2.7 Hz ,1H), 6.40 (dd, *J* = 9.1 Hz, 3.1 Hz ,1H), 4.76-4.66 (m, 2H), 2.91 (s, 3H), 2.04-1.95 (m, 2H), 1.03 (t, *J* = 7.1 Hz, 3H).

**^13^C-NMR (126 MHz, DMSO-*d*_6_):** δ = 155.1, 153.8, 153.2, 150.8, 150.2, 137.7, 137.7, 130.2, 130.2, 128.5, 128.3, 127.7, 127.3, 124.2, 123.4, 119.9, 119.5, 119.1, 119.0, 114.8, 114.4, 114.3, 114.1, 110.1, 110.0, 109.9, 109.4, 108.0, 108.0, 107.7, 106.4, 53.20, 44.54, 22.27, 11.39.

***R*_f_:** 0.25 (CH_2_Cl_2_**/***n*-heptane 5:1).

**Melting Point:** 255 – 257 ^o^C.

**HRMS (MALDI-FT-ICR):** Calculated for C_36_H_25_NO_4_ [M**^•^**^+^] 534.1700 found 535.1774.

Helicene **3c**:

Yield: 80 mg, 145 μmol, 5%.

**^1^H NMR (500 MHz, DMSO-*d*_6_):** δ = 8.56 (s, 2H), 7.95 (d, *J* = 8.81 Hz, 2H ), 7.89 (d, *J* = 8.81 Hz, 2H ), 7.48-7.44 (m, 4H), 7.40 (d, , *J* = 2.4 Hz, 2H) ,7.26 (d, *J* = 8.7 Hz, 2H), 6.36 (dd, *J* = 8.7, 2.4 Hz, 2H), 4.75-4.65 (m, 2H), 2.02 – 1.91 (m, 2H), 1.00 (t, *J* = 7.3 Hz, 3H).

**^13^C NMR (126 MHz, DMSO-*d*_6_):** δ = 153.7, 153.7, 150.7, 137.6, 130.5, 123.4, 119.4, 119.4, 114.5, 128.2, 127.3, 114.5, 109.9, 109.9, 108.4, 107.4, 44.43, 22.22, 11.50.

***R*_f_:** 0.3 (EtOAc/*n-*heptane 2:3).

**Melting Point:** Decompose 250 ^o^C.

**HRMS (MALDI-FT-ICR):** Calculated for C_35_H_23_NO_4_ [M**^•^**^+^] 521.1622 found 521.1685.

Synthesis of helicene **3a**

**Procedure:** In a 100 mL flame dried round bottom flask 3,6-dihydroxy-2,7-di-*tert*-butyl-N-propyl carbazole **1**, (200 mg, 0.565 mmol, 1.00 eq.) and 2-hydroxy-7-methoxy naphthalene **2**, (197 mg, 1.13 mmol, 2.00 eq.) were dissolved in anhydrous chlorobenzene (20 mL). Chloranil (278 mg, 1.13 mmol, 2.00 eq.) was added in one portion and the reaction mixture was stirred at room temperature under a nitrogen atmosphere for 7.5 hours. Then *p*-toluene sulfonic acid monohydrate (475 mg, 2.49 mmol, 4.40 eq.) was added and the reaction mixture was stirred under a nitrogen atmosphere at reflux for 69 hours. The reaction was quenched with saturated NaHCO_3_ (20 mL), water (20 mL) and brine (10 mL), thereafter extracted with CH_2_Cl_2_ (20 mL) and EtOAc (2×20 mL). The combined organic phases were concentrated *in vacuo* and purified by dry column vacuum chromatography (Ø: 2 cm, length: 6 cm, 20 mL fractions, toluene in *n*- heptane (v/v), 5% gradient increment, gradient held for 4 fractions at 50%, elutes in fractions 15 - 22) to yield helicene **3a** as a yellow solid.

Total yield calculated from the ^1^H-NMR spectrum of fraction 14 from the column, containing a mixture of helicene carrying one *tert*-butyl group and helicene **3a**.

**Yield:** 232 mg, 422 μmol, 74% (67% isolated).

Helicene **3a**: See experimental: Synthesis of helicenes **3a**, **3b** and **3c**.

Synthesis of helicene **3b**

**Procedure:** In a 50 mL flame-dried round bottom flask, helicene **3a** (400 mg, 727.7 μmol, 1 eq.) and tetrabutylammonium iodide (269 mg, 727.7 mmol, 1 eq.) were dissolved in anhydrous CH_2_Cl_2_ (12.5 mL) under an atmosphere of nitrogen. BCl_3_ (1.31 mL, 1.31 mmol, 1.8 eq., 1 m in CH_2_Cl_2_) was added dropwise through a syringe to the reaction mixture. The reaction mixture was stirred at room temperature under nitrogen for 3 hours and quenched by dropwise addition of water (10 mL) and thereafter extracted with dichloromethane (3×15 mL). The combined organic phases were concentrated *in vacuo* and purified by dry column vacuum chromatography (Ø: 3 cm, length: 6 cm, 20 mL fractions, CH_2_Cl_2_ in toluene (v/v), 5% gradient increment, desired compound elutes in fractions 14-20) to yield the product helicene **3b** as a yellow solid.

**Yield:** 127 mg, 237,1 μmol, 33%.

Helicene **3b**: See experimental: Synthesis of helicenes **3a**, **3b** and **3c**.

Synthesis of helicene **3c**

**Procedure:** In a 50 mL flame dried round bottom helicene **3a** (150 mg, 272.91 μmol, 1 eq.) and tetrabutylammonium iodide (403 mg, 1.09 mmol, 4 eq.) were dissolved in anhydrous CH_2_Cl_2_ (10 mL) under an atmosphere of nitrogen. BCl_3_ (2.73 mL, 2.73 mmol, 10 eq., 1 m in CH_2_Cl_2_) was added dropwise through a syringe to the reaction mixture. The reaction mixture was stirred at room temperature for 3 hours and quenched by dropwise addition of water (10 mL) and extracted with dichloromethane (3×15 mL). The combined organic phases were concentrated *in vacuo* and purified by dry column vacuum chromatography (Ø: 2 cm, length: 6 cm, 20 mL fractions, CH_2_Cl_2_ in toluene (v/v), 15% gradient increment, elutes in fractions 9-12) to yield helicene **3c** as a yellow solid.

**Yield:** 100 mg, 191.7 μmol, 70%.

Helicene **3c**: See experimental: Synthesis of helicenes **3a**, **3b** and **3c**.

Synthesis of helicene **4**

**Procedure:** In a 100 mL round-bottom flask equipped with a septum helicene **3b** (120 mg, 224 μmol, 1 eq.) and compound **2** (132.7 mg, 762 μmol, 3.4 eq) were dissolved in chlorobenzene (60 mL). Cu^II^Cl(OH)-TMEDA (130 mg, 560 μmol, 2.50 eq.) was added in one portion. Two needles (0.8 × 40 mm) were pierced through the septum. The reaction mixture was stirred at room temperature for 16 hours. The reaction mixture was filtered through a silica plug (5 cm, Ø : 3 cm) eluted with EtOAc (200 mL). The mixture was concentrated *in vacuo* and redissolved in chlorobenzene (50 mL). *p*-Toluene sulfonic acid monohydrate (426 mg, 2.24 mmol, 10 eq.) was added in one portion and the reaction mixture was heated to 100 ^o^C for 6 hours under a nitrogen atmosphere. The reaction was quenched by addition of water (100 mL) and extracted with CH_2_Cl_2_ (3×50 mL). The combined organic phases were concentrated *in vacuo* and purified by dry column vacuum chromatography (Ø : 3 cm, length: 7 cm, 35 mL fractions, EtOAc in *n*-heptane (v/v), 5% gradient increment) Fractions 7-10 were combined and further purified by dry column vacuum chromatography (Ø : 3 cm, length: 7 cm, 35 mL fractions, CH_2_Cl_2_ in *n*-heptane (v/v), 7.5% gradient increment, elutes in fractions 11) to yield helicene **4** as a yellow solid.

**Yield:** 29 mg, 41.0 μmol, 18%.

**^1^H-NMR (500 MHz, CD_2_Cl_2_):** δ = 7.85 (d, *J* = 8.7 Hz, 1H), 7.73 – 7.67 (m, 2H), 7.56 –7.52 (m, 2H) ,7.47 – 7.40 (m, 2H), 7.32 (dd, *J* = 8.7, 2.8 Hz, 2H), 7.28 – 7.21 (m, 2H), 7.15 (d, *J* = 2.6 Hz, 1H), 7.12 (d, *J* = 8.6 Hz, 1H), 6.98 (d, *J* = 8.6 Hz, 1H), 6.90 (d, *J* = 8.7 Hz, 1H), 6.73 (d, *J* = 2.6 Hz, 1H), 6.53 (dd, *J* = 8.8, 2.6 Hz, 1H), 6.50 (dd, *J* = 8.8, 2.6 Hz, 1H), 4.23 – 4.10 (m, 2H), 2.81 (s, 3H), 2.74 (s, 3H), 1.89 (app sext, *J* = 7.4 Hz, 2H), 1.11 (t, *J* = 7.4 Hz, 3H).

**^13^C-NMR (126 MHz, CD_2_Cl_2_):** δ = 155.8, 155.5, 155.3, 154.1, 153.8, 152.5, 150.7, 150.2, 138.0, 137.3, 130.2, 130.1, 129.5, 129.0, 128.3, 128.3, 127.7, 127.3, 126.9, 125.4, 125.1, 124.7, 123.2, 121.4, 120.9, 119.8, 119.8, 119.3, 119.2, 118.0, 115.9, 115.6, 114.9, 111.0, 110.6, 110.2, 110.2, 109.7, 109.2, 108.3, 108.0, 106.2, 105.9, 105.8, 54.28, 53.91, 45.74, 23.02, 12.20.

**Rf:** 0.54 (30% EtOAc in /*n*-heptane)

**HRMS (ESP-FT-ICR):** Calculated for C_47_H_31_NO_5_ [M+H^+^] 690.2275 found 690.2236.

Synthesis of helicene **5**

**Procedure:** In a 250 mL round-bottom flask equipped with a septum helicene **3c** (50.00 mg, 95.86 μmol, 1 eq.) and compound **2** (75.15 mg, 431.39 μmol, 4.5 eq) were dissolved in chlorobenzene (55 mL). Cu^II^Cl(OH)-TMEDA (49.64 mg, 213.78 μmol, 2.23 eq.) was added in one portion. Two needles (0.8 × 40 mm) were pierced through the septum. The reaction mixture was stirred at room temperature for 22 hours, then additional amount of compound **2** (50.1 mg, 287.5 μmol, 3 eq.) was added in one portion. The reaction was stirred 24 hours, quenched by addition of water (85 mL) and extracted with CH_2_Cl_2_ (3×65 mL). The combined organic phases were washed with water (65 mL) and concentrated *in vacuo*. The remaining solid was redissolved in chlorobenzene (55 mL) and *p-*toluene sulfonic acid monohydrate (182.35 mg, 958.64 μmol, 10 eq.) was added in one portion. The reaction mixture was heated to 100 °C and stirred for 3 hours under a nitrogen atmosphere, thereafter quenched by addition of water (20 mL) and extracted with CH_2_Cl_2_ (4×20 mL). The combined organic phases were washed with brine (50 mL) and concentrated *in vacuo* and purified by flash column chromatography (Isocratic, 100% CH_2_Cl_2_). Impure fractions containing helicene **5** were combined and repurified by flash column chromatography (20% EtOAc in *n-*heptane) to yield helicene **5** as a yellow solid.

Yield: 5 mg, 6.02 μmol, 6%.

**^1^H-NMR (500 MHz, CD_2_Cl_2_):** δ = 7.77 (d, *J* = 8.6 Hz, 2H), 7.65 (d, *J* = 8.6 Hz, 2H), 7.55 (d, *J* = 8.6 Hz, 2H), 7.22 (d, *J* = 8.7 Hz, 2H), 7.18 – 7.14 (m, 6H), 7.07 (d, *J* = 8.7 Hz, 2H), 6.75 (d, *J* = 8.7 Hz, 2H), 6.38 (dd, *J* = 8.7, 2.5 Hz, 2H), 6.12 (d, *J* = 2.5 Hz, 2H), 3.86 – 3.78 (m, 1H), 3.68 – 3.60 (m, 1H), 2.33 (s, 6H) 1.74 (app sext, *J* = 7.6 Hz, 2H), 1.10 (t, *J* = 7.6 Hz, 3H).

**^13^C-NMR (126 MHz, CD_2_Cl_2_):** δ = 154.8, 154.7, 154.1, 152.1, 149.8, 136.8, 130.0, 129.1, 128.1, 128.0, 127.8, 127.5, 124.5, 124.5, 121.6, 121.1, 119.9, 118.5, 118.4, 115.3, 110.9, 110.6, 109.3, 107.9, 106.3, 105.8, 53.92, 45.47, 22.05, 12.31.

**Rf:** 0.82 (100% CH_2_Cl_2_).

**HRMS (ESP-FT-ICR):** Calculated for C_36_H_23_NO_4_ [M+H^+^] 830.25371 found 830.25669.

# 3. NMR and MS Spectra (Figure SI 1 - 28)

**Figure SI 1.** ^1^H-NMR spectrum of helicene **3a** in DMSO-d_6_ (500 MHz).

**Figure SI 2.** ^13^C-spectrum of helicene **3a** in DMSO-d_6_ (126 MHz).

**Figure SI 3.** ^1^H-^1^H COSY spectrum of helicene **3a** in DMSO-d_6_ (500 MHz).

**Figure SI 4.**^1^H-^13^C HSQC spectrum of helicene **3a** in DMSO-d_6_ (500/126 MHz).

**Figure SI 5.** ^1^H-^13^C HMBC spectrum of helicene **3a** in DMSO-d_6_ (500/126 MHz)**.**

**Figure SI 6**. HRMS (MALDI^+^ FT-ICR) spectrum of helicene **3a**.

**Figure SI 7.** ^1^H-NMR spectrum of helicene **3b** in DMSO-d_6_ (500 MHz).

**Figure SI 8.** ^13^C-APT spectrum of **3b** in DMSO-d_6_ (126 MHz).

**Figure SI 9.** ^1^H-^1^H COSY spectrum of helicene **3b** in DMSO-d_6_ (500 MHz).

**Figure SI 10.** ^1^H-^13^C HSQC spectrum of helicene **3b** in DMSO-d_6_ (500/126 MHz)**.**

**Figure SI 11.** HRMS (MALDI^+^ FT-ICR) spectrum of helicene **3b.**

**Figure SI 12.** ^1^H-NMR spectrum of helicene **3c** in DMSO-d_6_ (500 MHz).

**Figure SI 13.** ^13^C-APT spectrum of helicene **3c** in DMSO-d_6_ (126 MHz).

**Figure SI 14.** ^1^H-^1^H COSY spectrum of helicene **3c** in DMSO-d_6_ (500 MHz).

Figure SI 15. ^1^H-^13^C COSY spectrum of helicene **3c** in DMSO-d_6_ (500/126 MHz).

Figure SI 16. HRMS (MALDI^+^ FT-ICR) spectrum of helicene **3c.**

**Figure SI 17.** ^1^H-NMR spectrum of helicene **4** in CD_2_Cl_2_ (500 MHz).

**Figure SI 18.** ^13^C-APT spectrum of helicene **4** in CD_2_Cl_2_ (500 MHz).

**Figure SI 19.** ^1^H-^1^H COSY spectrum of helicene **4** in CD_2_Cl_2_ (500 MHz).

**Figure SI 20.** ^1^H-^13^C HSQC spectrum of helicene **4** in CD_2_Cl_2_ (500/126 MHz)**.**

**Figure SI 21.** ^1^H-^13^C HMBC spectrum of helicene **4** in CD_2_Cl_2_ (500/126 MHz)**.**

**Figure SI 22.** HRMS (ESP^+^ FT-ICR) spectrum of helicene **4.**

**Figure SI 23.** ^1^H-NMR spectrum of helicene **5** in CD_2_Cl_2_ (500 MHz).

**Figure SI 24.** ^13^C-APT spectrum of helicene **5** in CD_2_Cl_2_ (500 MHz).

**Figure SI 25.** ^1^H-^1^H COSY spectrum of helicene **5** in CD_2_Cl_2_ (500 MHz).

**Figure SI 26.** ^1^H-^13^C HSQC spectrum of helicene **5** in CD_2_Cl_2_ (500/126 MHz)**.**

**Figure SI 27.** ^1^H-^13^C HMBC spectrum of helicene **4** in CD_2_Cl_2_ (500/126 MHz)**.**

**Figure SI 28.** HRMS (ESP^+^ FT-ICR) spectrum of helicene **5.**

# 4. X-Ray Crystallography

**Table SI 1.** Centroid to centroid distance of helicenes **3a**, **3b**, **3c**, **4**, and **5** measured from the terminal-to-terminal ring **A** for helicenes to ring **I**.

| Helicene | **3a** | **3b** | **3c** | **4** | **5** |
| --- | --- | --- | --- | --- | --- |
| Distance from  centroid to centroid | 3.869 Å  A to I | 3.852 Å  A to I | 3.825 Å  A to I | 3.621 Å  A to I | 3.573 Å  A to I |

**Figure SI 29.** X-ray crystal structures of helicenes **3a**, **4** and **5** with ring labeling. Methoxy and propyl groups omitted.

**Table SI 2.** Torsion angles (^o^) of helicene **3a**, **3b**, **3c**, **4** and **5** measured from the crystal structures.

| Ring | **3a** | **3b** | **3c** | **4** | **5** |
| --- | --- | --- | --- | --- | --- |
| B | 10.1 | 7.31 | 5.91 | 9.22 | 16.4 |
| C | 12.7 | 12.2 | 12.3 | 13.0 | 5.06 |
| D | 11.7 | 10.8 | 13.5 | 12.8 | 13.3 |
| E | 16.8 | 17.5 | 15.1 | 20.8 | 24.5 |
| F |  | 14.0 |  | 11.7 |  |
| G |  | 11.3 |  | 4.18 |  |
| H |  | 6.83 |  | 20.7 |  |
| I |  |  |  | 22.0 | 16.4 |
| J |  |  |  | 8.58 | 20.9 |
| K |  |  |  | 6.06 | 5.25 |

## 4.1. Helicene 3a

A yellow, Prism shaped crystal of helicene **3a** was mounted on a MiTeGen micromount with perfluoroether oil. The sample was crystallized by slow evaporation of a mixture of MeCN and n-heptane. Data were collected from a shock-cooled single crystal at 100(2) K on a Bruker D8 VENTURE dual wavelength Mo/Cu three-circle diffractometer with a microfocus sealed X-ray tube using a mirror optics as monochromator and a Bruker PHOTON III detector. The diffractometer was equipped with an Oxford Cryostream 800 low temperature device and used Mo*K_α_* radiation (λ = 0.71073 Å). All data were integrated with SAINT and a multi-scan absorption correction using SADABS was applied.^[48,49]^ The structure was solved by direct methods using SHELXT and refined by full-matrix least-squares methods against *F*^2^ by SHELXL-2019/1.^[50,51]^ The structure was refined as a 2-component inversion twin. All non-hydrogen atoms were refined with anisotropic displacement parameters. All hydrogen atoms were refined isotropic on calculated positions using a riding model with their *U*_iso_ values constrained to 1.5 times the *U*_eq_ of their pivot atoms for terminal sp^3^ carbon atoms and 1.2 times for all other carbon atoms. Crystallographic data for the structures reported in this paper have been deposited with the Cambridge Crystallographic Data Centre.^[52]^ CCDC 2301877 contain the supplementary crystallographic data for this paper. These data can be obtained free of charge from The Cambridge Crystallographic Data Centre via www.ccdc.cam.ac.uk/structures. This report and the CIF file were generated using FinalCif.^[53]^

| CCDC number | 2301877 |
| --- | --- |
| Empirical formula | C_37_H_27_NO_4_ |
| Formula weight | 549.59 |
| Temperature [K] | 100(2) |
| Crystal system | Orthorhombic |
| Space group (number) | $P2_{1}2_{1}2_{1}$ (19) |
| *a* [Å] | 16.1864(6) |
| *b* [Å] | 17.2580(5) |
| *c* [Å] | 19.5339(7) |
| α [°] | 90 |
| β [°] | 90 |
| γ [°] | 90 |
| Volume [Å^3^] | 5456.7(3) |
| *Z* | 8 |
| *ρ*_calc_ [gcm^−3^] | 1.338 |
| *μ* [mm^−1^] | 0.087 |
| *F*(000) | 2304 |
| Crystal size [mm^3^] | 0.768×0.252×0.139 |
| Crystal colour | Yellow |
| Crystal shape | Prism |
| Radiation | Mo*K_α_* (λ=0.71073 Å) |
| 2θ range [°] | 4.03 to 54.97 (0.77 Å) |
| Index ranges | −21 ≤ h ≤ 20 −22 ≤ k ≤ 22 −25 ≤ l ≤ 25 |
| Reflections collected | 52094 |
| Independent reflections | 12503 *R*_int_ = 0.1191 *R*_sigma_ = 0.0983 |
| Completeness to  θ = 25.242° | 99.9 % |
| Data / Restraints / Parameters | 12503/0/764 |
| Goodness-of-fit on *F*^2^ | 1.016 |
| Final *R* indexes  [*I*≥2σ(*I*)] | *R*_1_ = 0.0562 w*R*_2_ = 0.1191 |
| Final *R* indexes  [all data] | *R*_1_ = 0.0905 w*R*_2_ = 0.1354 |
| Largest peak/hole [eÅ^−3^] | 0.27/-0.26 |
| Flack X parameter | 0.4(13) |

## 4.2. Helicene 3b

A yellow, plate shaped crystal of helicene **3b** was mounted on a MiTeGen micromount with perfluoroether oil. The sample was crystallized slow evaporation from acetone. Data were collected from a shock-cooled single crystal at 100(2) K on a Bruker D8 VENTURE dual wavelength Mo/Cu three-circle diffractometer with a microfocus sealed X-ray tube using a mirror optics as monochromator and a Bruker PHOTON III detector. The diffractometer was equipped with an Oxford Cryostream 800 low temperature device and used Mo*K_α_* radiation (λ = 0.71073 Å). All data were integrated with SAINT and a multi-scan absorption correction using SADABS was applied.^[4,5]^ The structure was solved by direct methods using SHELXT and refined by full-matrix least-squares methods against *F*^2^ by SHELXL-2019/1.^[50,51]^ All non-hydrogen atoms were refined with anisotropic displacement parameters. All C-bound hydrogen atoms were refined isotropic on calculated positions using a riding model with their *U*_iso_ values constrained to 1.5 times the *U*_eq_ of their pivot atoms for terminal sp^3^ carbon atoms and 1.2 times for all other carbon atoms. Crystallographic data for the structures reported in this paper have been deposited with the Cambridge Crystallographic Data Centre.^[52]^ CCDC 2301878 contain the supplementary crystallographic data for this paper. These data can be obtained free of charge from The Cambridge Crystallographic Data Centre via www.ccdc.cam.ac.uk/structures. This report and the CIF file were generated using FinalCif.^[53]^

| CCDC number | 2301878 |
| --- | --- |
| Empirical formula | C_39_H_30_NO_5_ |
| Formula weight | 592.64 |
| Temperature [K] | 100(2) |
| Crystal system | Orthorhombic |
| Space group (number) | $Pca2_{1}$ (29) |
| *a* [Å] | 10.764(5) |
| *b* [Å] | 17.548(6) |
| *c* [Å] | 15.536(3) |
| α [°] | 90 |
| β [°] | 90 |
| γ [°] | 90 |
| Volume [Å^3^] | 2934.5(18) |
| *Z* | 4 |
| *ρ*_calc_ [gcm^−3^] | 1.341 |
| *μ* [mm^−1^] | 0.089 |
| *F*(000) | 1244 |
| Crystal size [mm^3^] | 0.250×0.200×0.030 |
| Crystal colour | yellow |
| Crystal shape | plate |
| Radiation | Mo*K_α_* (λ=0.71073 Å) |
| 2θ range [°] | 4.44 to 60.39 (0.71 Å) |
| Index ranges | −13 ≤ h ≤ 13 −23 ≤ k ≤ 22 −21 ≤ l ≤ 17 |
| Reflections collected | 18327 |
| Independent reflections | 6220 *R*_int_ = 0.0474 *R*_sigma_ = 0.0538 |
| Completeness to  θ = 25.242° | 99.7 % |
| Data / Restraints / Parameters | 6220/1/412 |
| Goodness-of-fit on *F*^2^ | 1.030 |
| Final *R* indexes  [*I*≥2σ(*I*)] | *R*_1_ = 0.0495 w*R*_2_ = 0.0990 |
| Final *R* indexes  [all data] | *R*_1_ = 0.0720 w*R*_2_ = 0.1090 |
| Largest peak/hole [eÅ^−3^] | 0.34/-0.29 |
| Flack X parameter | 0.4(14) |

## 4.3. Helicene 3c

Slow evaporation from MeCN/CH2Cl2. A clear yellowish yellow, prism-shaped crystal was mounted on the goniometer. Data for helicene **3c** were collected from a shock-cooled single crystal at 100.00 K on a Bruker D8 VENTURE dual wavelength Mo/Cu three-circle diffractometer with a microfocus sealed X-ray tube using a mirror optics as monochromator and a Bruker PHOTON III detector. The diffractometer used Mo*K_α_* radiation (λ = 0.71073 Å). All data were integrated with SAINT V8.40B and a multi-scan absorption correction using SADABS 2016/2 was applied.^[48,49]^ The structure was solved by dual methods with SHELXT 2018/2 and refined by full-matrix least-squares methods against *F*^2^ using SHELXL 2018/3.^[50,51]^ All non-hydrogen atoms were refined with anisotropic displacement parameters. All C-bound hydrogen atoms were refined isotropic on calculated positions using a riding model with their *U*_iso_ values constrained to 1.5 times the *U*_eq_ of their pivot atoms for terminal sp^3^ carbon atoms and 1.2 times for all other carbon atoms. Crystallographic data for the structures reported in this paper have been deposited with the Cambridge Crystallographic Data Centre.^[52]^ CCDC 2447429 contain the supplementary crystallographic data for this paper. These data can be obtained free of charge from The Cambridge Crystallographic Data Centre via www.ccdc.cam.ac.uk/‌structures. This report and the CIF file were generated using FinalCif.^[53]^

| CCDC number | 2447429 |
| --- | --- |
| Empirical formula | C_39_H_29_N_3_O_4_ |
| Formula weight | 603.65 |
| Temperature [K] | 100.00 |
| Crystal system | orthorhombic |
| Space group (number) | $Pca2_{1}$ (29) |
| *a* [Å] | 10.8979(7) |
| *b* [Å] | 17.6325(12) |
| *c* [Å] | 15.2425(10) |
| α [°] | 90 |
| β [°] | 90 |
| γ [°] | 90 |
| Volume [Å^3^] | 2929.0(3) |
| *Z* | 4 |
| *ρ*_calc_ [gcm^−3^] | 1.369 |
| *μ* [mm^−1^] | 0.090 |
| *F*(000) | 1264 |
| Crystal size [mm^3^] | 0.1×0.2×0.5 |
| Crystal colour | clear yellowish yellow |
| Crystal shape | Prism |
| Radiation | Mo*K_α_* (λ=0.71073 Å) |
| 2θ range [°] | 4.39 to 61.12 (0.70 Å) |
| Index ranges | −15 ≤ h ≤ 15 −25 ≤ k ≤ 25 −21 ≤ l ≤ 21 |
| Reflections collected | 36662 |
| Independent reflections | 8910  *R*_int_ = 0.0875 *R*_sigma_ = 0.0728 |
| Completeness to θ = 25.242° | 99.8 % |
| Data / Restraints / Parameters | 8910 / 1 / 422 |
| Absorption correction T_min_/T_max_ (method) | 0.6630 / 0.7461  (multi-scan) |
| Goodness-of-fit on *F*^2^ | 1.034 |
| Final *R* indexes  [*I*≥2σ(*I*)] | *R*_1_ = 0.0499 w*R*_2_ = 0.1119 |
| Final *R* indexes  [all data] | *R*_1_ = 0.0816 w*R*_2_ = 0.1268 |
| Largest peak/hole [eÅ^−3^] | 0.30/−0.20 |
| Flack X parameter | -0.8(7) |

## 4.4. Helicene 4

From isopropanol/DCM. A clear light yellow, block-shaped crystal was mounted on the goniometer. Data for helicene **4** was collected from a shock-cooled single crystal at 100.00 K on a Bruker D8 VENTURE dual wavelength Mo/Cu three-circle diffractometer with a microfocus sealed X-ray tube using a mirror optics as monochromator and a Bruker PHOTON III detector.The diffractometer used Mo*K_α_* radiation (λ = 0.71073 Å). All data were integrated with SAINT V8.40B and a multi-scan absorption correction using SADABS 2016/2 was applied.^[48,49]^ The structure was solved by dual methods with SHELXT 2018/2 and refined by full-matrix least-squares methods against *F*^2^ using XL.^[50,51]^ All non-hydrogen atoms were refined with anisotropic displacement parameters. All hydrogen atoms were refined isotropic on calculated positions using a riding model with their *U*_iso_ values constrained to 1.5 times the *U*_eq_ of their pivot atoms for terminal sp^3^ carbon atoms and 1.2 times for all other carbon atoms. Crystallographic data for the structures reported in this paper have been deposited with the Cambridge Crystallographic Data Centre.^[52]^ CCDC 2497017 contain the supplementary crystallographic data for this paper. These data can be obtained free of charge from The Cambridge Crystallographic Data Centre via www.ccdc.cam.ac.uk/‌structures. This report and the CIF file were generated using FinalCif.^[53]^

| CCDC number | 2497017 |
| --- | --- |
| Empirical formula | C_47_H_31_NO_5_ |
| Formula weight | 689.73 |
| Temperature [K] | 100.00 |
| Crystal system | orthorhombic |
| Space group (number) | $P2_{1}2_{1}2_{1}$ (19) |
| *a* [Å] | 11.3836(11) |
| *b* [Å] | 12.4098(13) |
| *c* [Å] | 23.076(3) |
| α [°] | 90 |
| β [°] | 90 |
| γ [°] | 90 |
| Volume [Å^3^] | 3259.9(6) |
| *Z* | 4 |
| *ρ*_calc_ [gcm^−3^] | 1.405 |
| *μ* [mm^−1^] | 0.091 |
| *F*(000) | 1440 |
| Crystal size [mm^3^] | 0.03×0.05×0.05 |
| Crystal colour | clear light yellow |
| Crystal shape | block |
| Radiation | Mo*K_α_* (λ=0.71073 Å) |
| 2θ range [°] | 3.73 to 50.05 (0.84 Å) |
| Index ranges | −13 ≤ h ≤ 13 −14 ≤ k ≤ 14 −27 ≤ l ≤ 27 |
| Reflections collected | 26557 |
| Independent reflections | 5756  *R*_int_ = 0.0909 *R*_sigma_ = 0.0807 |
| Completeness to θ = 25.025° | 99.9 % |
| Data / Restraints / Parameters | 5756 / 0 / 483 |
| Absorption correction T_min_/T_max_ (method) | 0.6735 / 0.7453  (multi-scan) |
| Goodness-of-fit on *F*^2^ | 1.011 |
| Final *R* indexes  [*I*≥2σ(*I*)] | *R*_1_ = 0.0446 w*R*_2_ = 0.0958 |
| Final *R* indexes  [all data] | *R*_1_ = 0.0678 w*R*_2_ = 0.1076 |
| Largest peak/hole [eÅ^−3^] | 0.37/−0.23 |
| Flack X parameter | -1.1(10) |

## 4.5. Helicene 5

A clear yellow, irregular-shaped crystal was mounted on the goniometer. Data for helicene **5** was collected from a shock-cooled single crystal at 100.0 K on a Bruker D8 Venture dual wavelength Mo/Cu three-circle diffractometer with a microfocus sealed X-ray tube using a mirror optics as monochromator and a Bruker PHOTON III detector. The diffractometer used Mo*K_α_* radiation (λ = 0.71073 Å). All data were integrated with SAINT V8.40B and a multi-scan absorption correction using SADABS 2016/2 was applied.^[48,49]^ The structure was solved by dual methods with ShelXT and refined by full-matrix least-squares methods against *F*^2^ using ShelXL.^[50,51]^ All non-hydrogen atoms were refined with anisotropic displacement parameters. All hydrogen atoms were refined with isotropic displacement parameters. Some of their coordinates were refined freely and some on calculated positions using a riding model with their *U*_iso_ values constrained to 1.5 times the *U*_eq_ of their pivot atoms for terminal sp^3^ carbon atoms and 1.2 times for all other carbon atoms. Crystallographic data for the structures reported in this paper have been deposited with the Cambridge Crystallographic Data Centre.^[52]^ CCDC 2497016 contain the supplementary crystallographic data for this paper. These data can be obtained free of charge from The Cambridge Crystallographic Data Centre via www.ccdc.cam.ac.uk/‌structures. This report and the CIF file were generated using FinalCif.^[53]^

| CCDC number | 2497016 |
| --- | --- |
| Empirical formula | C_57_H_34_NO_6_ |
| Formula weight | 828.85 |
| Temperature [K] | 100.0 |
| Crystal system | monoclinic |
| Space group (number) | $C2/c$ (15) |
| *a* [Å] | 17.4720(6) |
| *b* [Å] | 15.4836(5) |
| *c* [Å] | 14.5428(5) |
| α [°] | 90 |
| β [°] | 105.0760(10) |
| γ [°] | 90 |
| Volume [Å^3^] | 3798.8(2) |
| *Z* | 4 |
| *ρ*_calc_ [gcm^−3^] | 1.449 |
| *μ* [mm^−1^] | 0.094 |
| *F*(000) | 1724 |
| Crystal size [mm^3^] | 0.08×0.1×0.15 |
| Crystal colour | clear yellow |
| Crystal shape | irregular |
| Radiation | Mo*K_α_* (λ=0.71073 Å) |
| 2θ range [°] | 4.83 to 54.97 (0.77 Å) |
| Index ranges | −22 ≤ h ≤ 22 −20 ≤ k ≤ 20 −18 ≤ l ≤ 18 |
| Reflections collected | 43025 |
| Independent reflections | 4354  *R*_int_ = 0.0372 *R*_sigma_ = 0.0198 |
| Completeness to θ = 25.242° | 99.8 % |
| Data / Restraints / Parameters | 4354 / 4 / 320 |
| Absorption correction T_min_/T_max_ (method) | 0.7147 / 0.7459  (multi-scan) |
| Goodness-of-fit on *F*^2^ | 1.027 |
| Final *R* indexes  [*I*≥2σ(*I*)] | *R*_1_ = 0.0465 w*R*_2_ = 0.1185 |
| Final *R* indexes  [all data] | *R*_1_ = 0.0533 w*R*_2_ = 0.1249 |
| Largest peak/hole [eÅ^−3^] | 0.43/−0.55 |

# 5. Optical Measurements

## 5.1. Fluorescence lifetimes

**Figure SI 30**. Time-resolved fluorescence decay plot of helicene **3a** in CH_2_Cl_2_.

**Figure SI 31**. Time-resolved fluorescence decay plot of helicene **4** in CH_2_Cl_2_.

### 5.1.1. Helicene 5

**Figure SI 32.** Time-resolved fluorescence decay plot of helicene **5** in CH_2_Cl_2_.

## 5.2. Chiral Chromatography

**Figure SI 33**. Recycling chiral HPLC UV-chromatogram (300 nm) of helicene **5** on a Nacalai Tesque COSMOSIL cholester column.

**Figure SI 34**. Recycling chiral HPLC UV-chromatogram (254 nm) of helicene **4** on a Nacalai Tesque COSMOSIL cholester column.

**Figure SI 35.** Recycling chiral HPLC UV-chromatogram (254 nm) of helicene **3a** on a Nacalai Tesque COSMOSIL cholester column.

## 5.3. Quantum yield measurements.

**Figure SI 36.** Integrated emission as a function of absorption at 395 nm for coumarin-153 in EtOH utilized for the quantification fluorescence quantum yield.

**Figure SI 37.** Absorbance dilutions utilized for the quantification of the fluorescence quantum yield for coumarin-153 in EtOH.

**Figure SI 38**. Fluorescence dilutions utilized for the quantification of the coumarin fluorescence quantum yield in EtOH.

**Figure SI 39.** Integrated emission as a function of absorption at 395 nm for helicene **3a** in CH_2_Cl_2_ utilized for the quantification fluorescence quantum yield.

**Figure SI 40.** Fluorescence dilutions utilized for the quantification of the fluorescence quantum for helicene **3a** yield in CH_2_Cl_2_.

**Figure SI 41.** Absorbance dilutions utilized for the quantification of the fluorescence quantum yield for helicene **3a** in CH_2_Cl_2_.

**Figure SI 42**. Integrated emission as a function of absorption at 395 nm for helicene **4** in CH_2_Cl_2_ utilized for the quantification fluorescence quantum yield.

**Figure SI 43.** Absorbance dilutions utilized for the quantification of the fluorescence quantum yield for helicene **4** in CH_2_Cl_2_.

**Figure SI 44.** Fluorescence dilutions utilized for the quantification of the fluorescence quantum for helicene **4** yield in CH_2_Cl_2_.

**Figure SI 45.** Integrated emission as a function of absorption at 395 nm for helicene **5** in CH_2_Cl_2_ utilized for the quantification fluorescence quantum yield.

**Figure SI 46.** Absorbance dilutions utilized for the quantification of the fluorescence quantum yield for helicene **5** in CH_2_Cl_2_.

**Figure SI 47.** Fluorescence dilutions utilized for the quantification of the fluorescence quantum for helicene **5** yield in CH_2_Cl_2_.

## 5.4. Summary of Chiral Optical Properties

**Table SI 3**. Summary of the chiral optical properties of helicenes **3a**, **4** and **5**. All chiral optical measurements were recorded in CH_2_Cl_2_.

|  | $\lambda_{max}$(nm) | $\varepsilon$ (м^-1^·cm^-1^) | Stokes shift (cm^-1^) | F (%) | *g*_lum_ | *g*_abs_ |  |  |  | CPL-Brightness (м^-1^·cm^-1^) |
| --- | --- | --- | --- | --- | --- | --- | --- | --- | --- | --- |
| [9] | 420 | 24000 (±711) | 1338 | 51 | 1.94·10^-3^ | 1.84·10^-3^  (425 nm) |  |  |  | 11.87 |
| [12] | 429 | 13900 (±711) | 1380 | 18 | 1.28·10^-2^ | 4.89·10^-3^  (436 nm) |  |  |  | 16.01 |
| [15] | 438 | 6220 (±105) | 1778 | 10 | 1.95·10^-2^ | 8.51·10^-3^  (at 446 nm) |  |  |  | 6.06 |

**Table SI 4**. Radioactive and non-radiative rate constants.

|  | $k_{r}$ (ns^-1^) | $k_{nr}$ (ns^-1^) | F (%) | Fluorescence lifetime  t (ns) |  |  |  |
| --- | --- | --- | --- | --- | --- | --- | --- |
| [9] | 0.123 | 0.118 | 51 | 4.16 |  |  |  |
| [12] | 0.043 | 0.195 | 18 | 4.21 |  |  |  |
| [15] | 0.029 | 0.259 | 10 | 3.48 |  |  |  |

## 5.5. Literature *g*_lum_ Comparison

In order to compare the effect of elongation for different helical structures reported in literature we plotted the *g*lum value as a function of helical length. The structures are shown in **Figure SI 56** and the plot is shown in **Figure SI 57**.

**Figure SI 48.** Selected examples of homologues series of helicenes reported in literature: extended[n]helicenes, PDI-fused helicenes, expanded[n]helicenes, aza[n]helicenes and PDI-fused helicenes. Used in plot below.

**Figure SI 49.** g_lum_ As a function of ring count [n] for selected examples of homologous series of helicenes reported in literature. The number of rings for the PDI-helicenes are counted as the number of ortho-annulated rings as a measure of length, as previously described.^[54]^

# 6. Computational Details and Spectra

The *p*-enantiomers of the helicenes **3a**, **4,** and **5** were optimized in vacuum at the CAM-B3LYP-D4^[55,56]^/ def2-SVP^[57]^ level-of-theory with the def2/J auxiliary basis set as implemented in ORCA.^[58,59]^ All subsequent calculations were performed at the same level of theory. Solvated structures were obtained by reoptimizing the optimized vacuum structures in dichloromethane (CH_2_Cl_2_) using the Conductor-like Polarizable Continuum Model (C-PCM).^[60]^ Time-dependent Density Functional Theory (TD-DFT) calculations were performed for both vacuum and solvated structures, obtaining the 50 lowest lying singlet excitation energies and the associated oscillator strengths and rotatory strengths. Comparison of the oscillator strengths calculated using both the velocity gauge and the length gauge yielded closely matching values, indicating good basis set saturation. UV-Vis and ECD spectra were obtained by convolution to a Lorentzian with full width half maxima of 0.2 eV and 0.12398 eV for UV-Vis and ECD respectively.

Geometry optimizations on the first singlet excited states (S1) were performed, consistent with Kasha’s rule that emission occurs from the S1 state. Vibrational analysis of the ground-states (S0) and the excited states (S1) geometries was carried out to acquire the Hessians. Following this, the S1 🡪 S0 emission and CPL spectra were simulated with a temperature of 298.15K using the Adiabatic Hessian (AH) and Franck-Condon approximation as implemented in the ORCA excited-state-dynamics module.^[61,62]^ The broadening of the spectra was carried out using a Lorentzian with a full width half maximum of 0.012398 eV.

**Figure SI 50.** Top: Simulated fluorescence spectra for helicenes **3a**, **4** and **5** in CH_2_Cl_2_**.** Bottom: Simulated CPL spectra of helicenes **3a**, **4** and **5** in CH_2_Cl_2_.

**Figure SI 51.** Top: Simulated absorbance spectra of helicenes **3a**, **4** and **5** in CH_2_Cl_2_. Bottom: Simulated ECD spectra of helicenes **3a**, **4** and **5**.

**Table SI 5**. Calculated magnitude of magnetic |m| and electronic transition dipole moment |m| and the angle between them (q). au = atomic units. Values calculated in CH_2_Cl_2_, values in parentheses denote values calculated in vacuum.

| Helicene[n] | \|*m*\|(au) | \|*m*\|(au) | *q* (^o^) |
| --- | --- | --- | --- |
| [9] (**3a**) | 1.599 (1.632) | 1.672 (1.051) | 118.1 (132.8) |
| [12] (**4**) | 1.591 (1.600) | 1.301 (0.844) | 128.6 (141.5) |
| [15] (**5**) | 1.603 (1.638) | 1.169 (0.75523) | 138.9 (151.7) |

**Figure SI 52.** Calculated natural transition orbitals of helicenes in CH_2_Cl_2_ for S_0_-S_1_ transition. Left: Acceptor. Right: Donor. a) helicene **3a**, b) helicene **4**, c) helicene **5**.

# 7. References

[45]	C. Würth, M. Grabolle, J. Pauli, M. Spieles, U. Resch-Genger, “Relative and absolute determination of fluorescence quantum yields of transparent samples” *Nat Protoc* **2013**, *8*, 1535–1550.

[46]	C. B. Nielsen, T. Brock‐Nannestad, P. Hammershøj, T. K. Reenberg, M. Schau‐Magnussen, D. Trpcevski, T. Hensel, R. Salcedo, G. V. Baryshnikov, B. F. Minaev, M. Pittelkow, “Azatrioxa[8]circulenes: Planar Anti‐Aromatic Cyclooctatetraenes” *Chemistry A European J* **2013**, *19*, 3898–3904.

[47]	S. S. Warthegau, A. E. Hillers‐Bendtsen, S. K. Pedersen, C. Rindom, C. Bræstrup, J. S. Jensen, O. Hammerich, M. S. Thomsen, F. S. Kamounah, P. Norman, K. V. Mikkelsen, T. Brock‐Nannestad, M. Pittelkow, “Heterocyclic [9]Helicenes Exhibiting Bright Circularly Polarized Luminescence” *Chemistry A European J* **2023**, *29*, e202301815.

[48]	L. Krause, R. Herbst-Irmer, G. M. Sheldrick, D. Stalke, “Comparison of silver and molybdenum microfocus X-ray sources for single-crystal structure determination” *J Appl Crystallogr* **2015**, *48*, 3–10.

[49]	“Bruker, SAINT, V8.40B, Bruker AXS Inc., Madison, Wisconsin, USA,” **n.d.**

[50]	G. M. Sheldrick, “*SHELXT* – Integrated space-group and crystal-structure determination” *Acta Crystallogr A Found Adv* **2015**, *71*, 3–8.

[51]	G. M. Sheldrick, “Crystal structure refinement with *SHELXL*” *Acta Crystallogr C Struct Chem* **2015**, *71*, 3–8.

[52]	C. R. Groom, I. J. Bruno, M. P. Lightfoot, S. C. Ward, “The Cambridge Structural Database” *Acta Crystallogr B Struct Sci Cryst Eng Mater* **2016**, *72*, 171–179.

[53]	“D. Kratzert, FinalCif, V155, https://dkratzert.de/finalcif.html.,” **n.d.**

[54]	C. Bræstrup, X. Xiao, F. García‐González, T. Brock‐Nannestad, D. Aranda, S. T. Bao, D. Cavlovic, H. Jiang, F. Ng, C. Nuckolls, F. Santoro, M. Pittelkow, “Helical Ladder Oligomers Exhibit Amplified Circularly Polarized Emission” *Advanced Optical Materials* **2025**, *13*, 2403061.

[55]	T. Yanai, D. P. Tew, N. C. Handy, “A new hybrid exchange–correlation functional using the Coulomb-attenuating method (CAM-B3LYP)” *Chemical Physics Letters* **2004**, *393*, 51–57.

[56]	E. Caldeweyher, C. Bannwarth, S. Grimme, “Extension of the D3 dispersion coefficient model” *The Journal of Chemical Physics* **2017**, *147*, 034112.

[57]	F. Weigend, R. Ahlrichs, “Balanced basis sets of split valence, triple zeta valence and quadruple zeta valence quality for H to Rn: Design and assessment of accuracy” *Phys. Chem. Chem. Phys.* **2005**, *7*, 3297.

[58]	F. Neese, G. Olbrich, “Efficient use of the resolution of the identity approximation in time-dependent density functional calculations with hybrid density functionals” *Chemical Physics Letters* **2002**, *362*, 170–178.

[59]	F. Neese, “Software Update: The ORCA Program System—Version 6.0” *WIREs Comput Mol Sci* **2025**, *15*, e70019.

[60]	V. Barone, M. Cossi, “Quantum Calculation of Molecular Energies and Energy Gradients in Solution by a Conductor Solvent Model” *J. Phys. Chem. A* **1998**, *102*, 1995–2001.

[61]	B. De Souza, G. Farias, F. Neese, R. Izsák, “Predicting Phosphorescence Rates of Light Organic Molecules Using Time-Dependent Density Functional Theory and the Path Integral Approach to Dynamics” *J. Chem. Theory Comput.* **2019**, *15*, 1896–1904.

[62]	B. De Souza, F. Neese, R. Izsák, “On the theoretical prediction of fluorescence rates from first principles using the path integral approach” *The Journal of Chemical Physics* **2018**, *148*, 034104.
